# A Retrospective Analysis of the Association of Neutrophil–Lymphocyte Ratio (NLR) with Anemia in the Saudi Population

**DOI:** 10.3390/medicina59091592

**Published:** 2023-09-03

**Authors:** Yazeed Alshuweishi, Mohammed Alfaifi, Yousef Almoghrabi, Yazeed A. Al-Sheikh, Mohammad A. Alfhili

**Affiliations:** 1 Chair of Medical and Molecular Genetics Research, Department of Clinical Laboratory Sciences, College of Applied Medical Sciences, King Saud University, Riyadh 12372, Saudi Arabia; yalshuweishi@ksu.edu.sa (Y.A.); yalsheikh@ksu.edu.sa (Y.A.A.-S.); 2 Department of Clinical Laboratory Sciences, College of Applied Medical Sciences, King Khalid University, Abha 61421, Saudi Arabia; mhalfaifi@kku.edu.sa; 3 Department of Clinical Biochemistry, Faculty of Medicine, King Abdulaziz University, Jeddah 21589, Saudi Arabia; yalmoghrabi@kau.edu.sa; 4 Department of Clinical Pathology, Al Borg Diagnostics, Jeddah 23226, Saudi Arabia

**Keywords:** anemia, NLR, inflammation, Saudi Arabia

## Abstract

*Background*: The link between inflammation and anemia is well established but fluctuations in the emerging inflammatory index, neutrophil–lymphocyte ratio (NLR), in anemic subjects remain ambiguous. The purpose of this study is to address the prevailing knowledge gaps regarding the association of NLR with anemia in the Saudi population. *Methods*: Laboratory results of NLR, C-reactive protein (CRP), and hemoglobin for 14,261 subjects were obtained from Al Borg Diagnostics and retrospectively analyzed. Means, risk measures, and the diagnostic performance of NLR for anemia were examined in age- and gender-wise comparisons. *Results*: NLR was significantly elevated in anemic individuals and those with high NLR had a significantly lower Hb concentration. Moreover, elevated NLR was more prevalent in anemic subjects (PR: 1.87, 95% CI: 1.46–2.40, *p* < 0.0001) and carried a greater risk for the condition (OR: 1.91, 95% CI: 1.47–2.48, *p* < 0.0001) as did CRP. These observations demonstrated distinct age- and gender-specific patterns. However, both parameters were of no value in the diagnosis of anemia as seen from receiver operating characteristic curves. *Conclusions*: Altogether, these findings indicate that elevated NLR is associated with anemia, which suggests its usefulness for monitoring rather than diagnosing anemia associated with inflammation in Saudi subjects. Further examination of this association in longitudinal studies is needed.

## 1. Introduction

Anemia is defined as a condition in which the total number of circulating red blood cells (RBCs) is diminished or their oxygen-carrying capacity is inadequate to meet physiological needs [1]. Anemia can be diagnosed by a reduction in RBC count, hematocrit (HCT), or hemoglobin (Hb), with the latter being the most commonly used to define anemia [2]. It was reported that anemia affects 2.2 billion people around the world with a prevalence rate of 33% [3]. Anemia prevalence is highest in developing countries and is common at all stages of life [4]. Although both males and females of all ages are affected, the most vulnerable groups are pregnant women and young children. In non-pregnant women aged 15–49 years, the prevalence of anemia was 30%, while it was 36% in their pregnant counterparts [5]. The same study reported that anemia prevalence among children aged 6–59 months exceeded 70% in 11 countries [5]. In Saudi Arabia, it was shown that approximately 40% of women aged 15–49 years had anemia with an Hb concentration of <12 g/dL [6].

The most prevalent cause of anemia is iron deficiency (IDA), followed by anemia of chronic disease (ACD) [7]. ACD, also named anemia of inflammation, is seen in kidney disease, congestive heart failure, cancer, and autoimmune disorders [7] with concurrent elevated inflammatory markers [8]. Additionally, there was a link between inflammatory status and erythropoietin therapy resistance in anemic patients with chronic kidney disease [9]. This is likely due to the continuous increase in inflammatory response associated with the excessive spill of proinflammatory cytokines into the circulation. Although the exact mechanism is not fully understood, the inflammatory mediators eventually lead to reduced endogenous erythropoietin production, decreased erythroid progenitor sensitivity to erythropoietin, shortened erythrocyte lifespan, and reduced iron availability for hemoglobin synthesis despite sufficient body stores of iron [7].

A number of studies have identified blood-neutrophil-to-lymphocyte ratio (NLR) as an important marker of inflammation, which has significant prognostic implications in a number of disease states, particularly those that involve the cardiovascular, renal, and gastrointestinal systems. Binnetoglu et al. studied 69 Turkish patients with chronic renal failure and found that NLR correlated with proteinuria, demonstrating that NLR has a prognostic importance in this group of patients [10]. Moreover, NLR was a more sensitive parameter than increased white blood cell count in patients with acute appendicitis [11]. In a study involving a Saudi population, NLR ratio was found to be an important tool in determining COVID-19 clinical status [12]. NLR is also an emerging marker in patients with heart failure, acute coronary syndrome, hypertension, and diabetes [13,14,15,16,17].

NLR is a promising marker for the diagnosis and management of anemia in different settings of chronic disease, but population-based studies are very scarce. Furthermore, the effect of age and gender on the association between NLR and Hb remains understudied. Therefore, this study was aimed at evaluating blood NLR as an inflammatory marker in anemic individuals compared with normal controls.

## 2. Materials and Methods

### 2.1. Study Design and Data Collection

**Figure 1:**
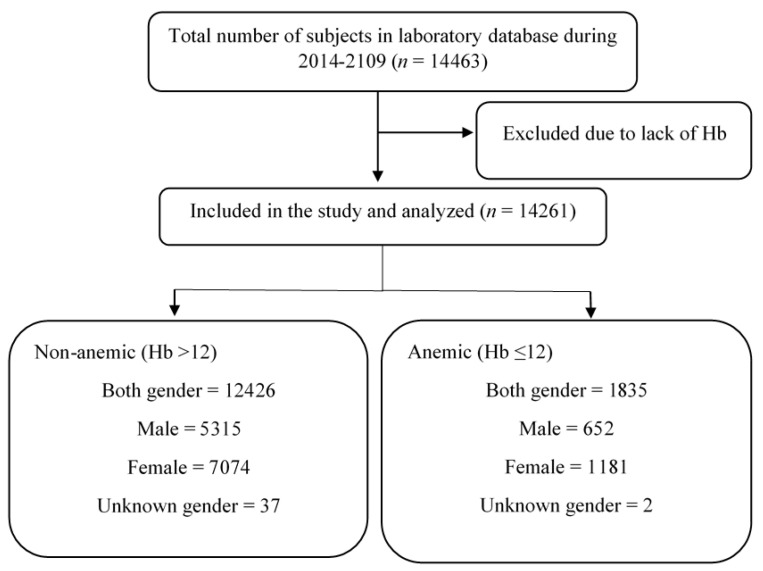
A flow chart of study design.

This is a retrospective study approved by the Biomedical Ethics Unit of Al Borg Diagnostics, Jeddah, Saudi Arabia (Reference No. 07/21, approved on 27 December 2021). Gender, age, and lab data for 14463 subjects collected from 2014 to 2019 were obtained from Al Borg Diagnostics database. Data were retrospectively analyzed, and the inclusion criteria were the presence of Hb and NLR in all age and sex groups. Beckman Coulter’s DxH 800 hematology analyzer (Brea, CA, USA) was used to obtain CBC values, while NLR was manually calculated. All records that did not include both variables were excluded from this study. Males and females were separated and age groups were formed based on our previous reports [18]. As shown in Table 1 and Figure 1, age groups were as follows: young (<18 years), young adults (18–39 years), adults (40–64 years), and elderlies (≥65 years). Anemia was defined by Hb level of ≤12 g/dL [6]. The normal range of NLR was set as 0.75–3.0 [19].

### 2.2. Statistics

GraphPad Prism v9.2.0 (GraphPad Software, Inc., San Diego, CA, USA) was used for analysis and statistical significance was set at a *p* value of <0.05. The data were not normally distributed, as revealed through D’Agostino and Pearson test and Kolmogorov–Smirnov test (*p* < 0.0001), and nonparametric tests were used for statistical analysis. Results were compared either using the unpaired, Mann–Whitney U test for two groups or Kruskal–Wallis test for three or more groups, and they are displayed as means ± confidence interval (95% CI) in figures and means ± standard error of the mean (SEM) in text. Association between Hb and NLR was tested through simple linear regression, and risk was assessed from the prevalence risk (PR) and odds ratio (OR). Sensitivity and specificity were examined through receiver operating characteristic (ROC) curve analysis and area under the curve (AUC) calculations.

## 3. Results

### 3.1. NLR Is Significantly Elevated in Anemic Individuals

**Figure 2:**
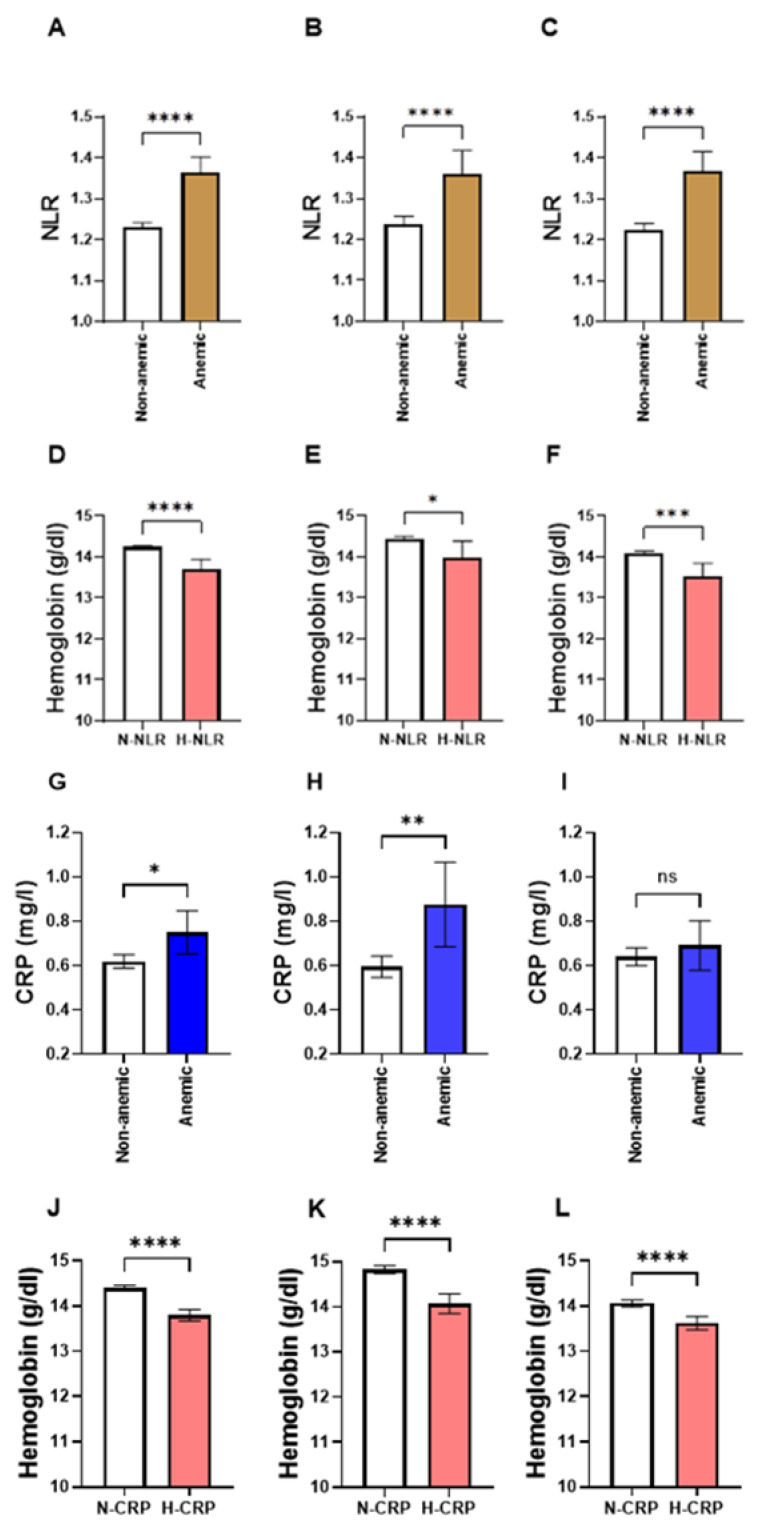
Comparison between NLR and CRP in light of Hb levels in males and females. Means ± 95% CI of NLR in non-anemics and anemics of (**A**) both genders, (**B**) males, and (**C**) females. Means ± 95%CI of Hb in normal NLR (N-NLR) and high NLR (H-NLR) groups in (**D**) both genders, (**E**) males, and (**F**) females. Means ± 95%CI of CRP concentrations in non-anemics and anemics of (**G**) both genders, (**H**) males, and (**I**) females. Means ± 95% CI of Hb concentrations in normal CRP (N-CRP) and high CRP (H-CRP) groups in (**J**) both genders, (**K**) males, and (**L**) females. ns indicates not significant while * (*p* < 0.05), ** (*p* < 0.01), *** (*p* < 0.001), and **** (*p* < 0.0001).

In order to assess NLR levels in light of anemia, subjects of both genders and across all age groups were defined as either anemic (hemoglobin level of ≤12 g/dL) or non-anemic (hemoglobin level of >12 g/dL). The total number of participants in our population was 14,261, of whom 12426 were non-anemic and 1835 were anemic. As demonstrated in Figure 2A, NLR was significantly increased in the anemic group (1.37, ±0.02) in comparison to the non-anemic group (1.23, ±0.01). This pattern was also true when males and females were analyzed separately. In Figure 2B, the male anemic group had higher levels of NLR (1.36, ±0.03), compared to the non-anemic male group (1.24, ±0.01). Similarly, NLR was significantly elevated in the female anemic group (Figure 2C; 1.37, ±0.02), compared to the non-anemic female group (Figure 2C; 1.22, ±0.01). On the other hand, CRP levels were significantly higher in the anemic group of both genders (Figure 2D; 0.62 ± 0.02 mg/dL vs. 0.75 ± 0.05 mg/dL) and in males (Figure 2E; 0.60 ± 0.02 mg/dL vs. 0.02 ± 0.10 mg/dL). However, the female anemic group exhibited a comparable level of CRP compared with the non-anemic female group, as shown in Figure 2F (0.64 ± 0.02 mg/dL vs. 0.69 ± 0.06 mg/dL).

### 3.2. Subjects with High NLR Have a Lower Hb Concentration

The examination of hemoglobin concentration in light of NLR levels further supported the association of anemia with NLR levels. Figure 2D–F show that the levels of hemoglobin (Hb) were consistently significantly reduced in the H-NLR group in both genders (Figure 2D; 14.22 ± 0.02 mg/dL vs. 13.70 ± 0.13 mg/dL), in males (Figure 2E; 14.42 ± 0.03 mg/dL vs. 13.97 ± 0.21 mg/dL), and in females (Figure 2F; 14.07 ± 0.03 mg/dL vs. 13.53 ± 0.16 mg/dL). Similarly, the H-CRP group had a lower Hb concentration when compared to the N-CRP group in both genders (Figure 2J; 14.40 ± 0.03 mg/dL vs. 13.79 ± 0.06 mg/dL), in males (Figure 2K; 14.83 ± 0.04 mg/dL vs. 14.07 ± 0.11 mg/dL), and in females (Figure 2L; 14.06 ± 0.04 mg/dL vs. 1362 ± 0.08 mg/dL)

### 3.3. Age-Specific Comparisons of NLR in Anemia

**Figure 3:**
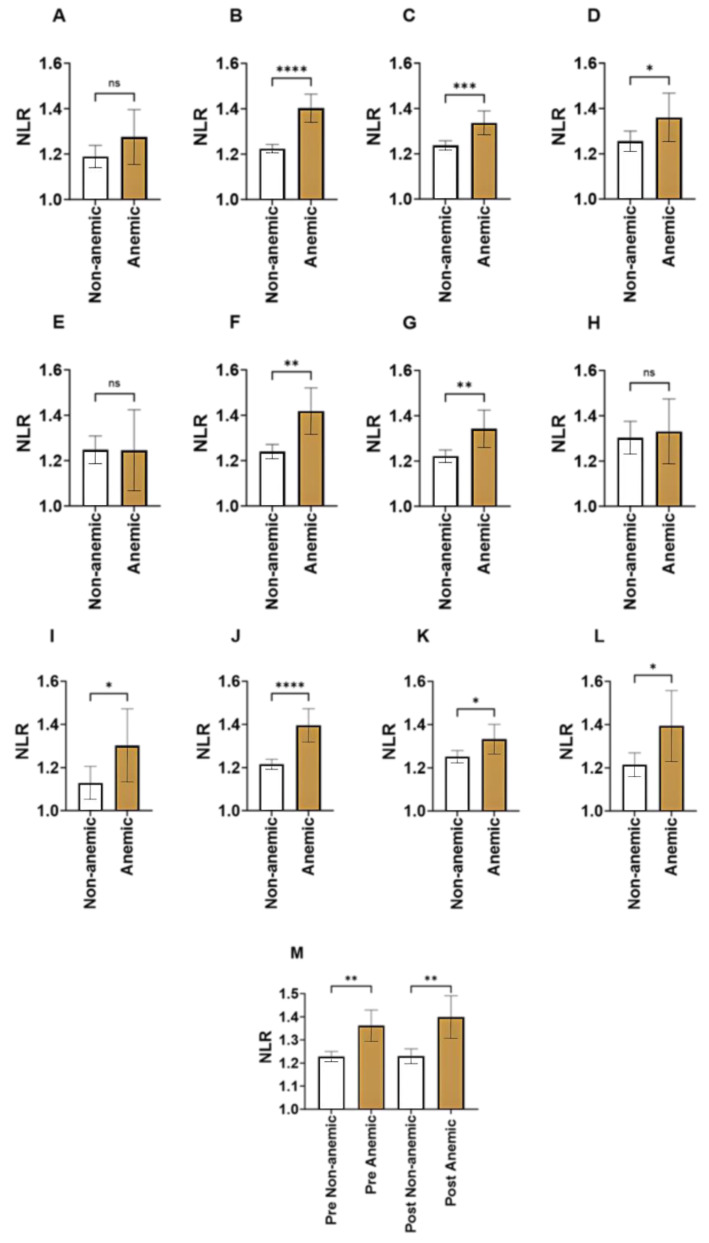
Gender- and age-wise comparisons of NLR in anemia. Means ± 95% CI of NLR in non-anemics and anemics. Both genders are shown for (**A**) young, (**B**) young adults, (**C**) adults, (**D**) and elderlies. Males are shown for (**E**) young, (**F**) young adults, (**G**) adults, and (**H**) elderlies. Females are shown for (**I**) young, (**J**) young adults, (**K**) adults, and (**L**) elderlies. (**M**) NLR values in premenopausal and postmenopausal anemic and non-anemic subjects. ns indicates not significant while * (*p* < 0.05), ** (*p* < 0.01), *** (*p* < 0.001), and **** (*p* < 0.0001).

The further stratification of study subjects by age demonstrated that NLR was significantly elevated in anemic young adults (Figure 3B; 1.22 ± 0.01 vs. 1.40 ± 0.03), anemic adults (Figure 3C; 1.24 ± 0.01 vs. 1.34 ± 0.03), and anemic elderlies (Figure 3D; 1.26 ± 0.02 vs. 1.36 ± 0.05) when compared to their normal counterparts. Only in the young group did NLR fail to distinguish between anemic and non-anemic, as depicted in Figure 3A (1.19 ± 0.02 vs. 1.28 ± 0.06). Furthermore, NLR was higher in anemic premenopausal (Figure 3M; 1.23 ± 0.01 vs. 1.36 ± 0.03) and postmenopausal woman (Figure 3M; 1.23 ± 0.02 vs. 1.40 ± 0.05) compared to the non-anemic counterparts. No significant difference in NLR was observed between premenopausal and postmenopausal anemics (Figure 3M; 1.36 ± 0.03 vs. 1.40 ± 0.05).

### 3.4. Gender-Wise Comparisons of NLR in Anemia

Combined age- and gender-controlled comparisons revealed that NLR was only elevated in young adults (Figure 3F; 1.24 ± 0.02 vs. 1.42 ± 0.05) and adults (Figure 3G; 1.22 ± 0.01 vs. 1.34 ± 0.04) of the anemic male groups. On the contrary, anemic females across all age groups had a higher level of NLR when compared to their normal counterparts (Figure 3I; 1.13 ± 0.04 vs. 1.30 ± 0.08; Figure 3J; 1.22 ± 0.01 vs. 1.40 ± 0.04; Figure 3K; 1.25 ± 0.01 vs. 1.33 ± 0.03; Figure 3L; 1.21 ± 0.03 vs. 1.39 ± 0.08)

### 3.5. Elevated NLR Is More Prevalent in Anemic Subjects

As demonstrated in Table 2, the prevalence of H-NLR among non-anemic subjects was 2.67%, and this rate increased in anemic subjects to 4.98%. Similarly, when males were analyzed alone, H-NLR was more prevalent in the anemic (4.46%) compared to non-anemic group (2.37%). A more profound pattern of increase was observed in female subjects where the prevalence of H-NLR increased from 2.91% to 5.28%. While the prevalence of elevated CRP increased from 13.27% to 24.63% in males, the female group showed a modest increase in H-CRP prevalence (16.67% to 19.07).

Moreover, Table 3 shows that a high level of NLR was associated with an increased risk of anemia in both genders (PR = 1.87, 95% CI: 1.46–2.40, *p* < 0.0001), in males (PR = 1.88, 95% CI: 1.23–2.89, *p* = 0.004), and in females (PR = 1.81, 95% CI: 1.34–2.46, *p* = 0.0001). Additionally, H-NLR in males or females was 1.92 and 2.22 times more likely to fall into the anemic group, respectively.

On the other hand, elevated CRP concentration was only associated with an increased risk of anemia in males but not females (Table 4), demonstrating that NLR performed better than CRP as an early predicator of anemia, particularly in females.

Elevated NLR but not CRP is associated with an increased risk of anemia in female subjects.

**Figure 4:**
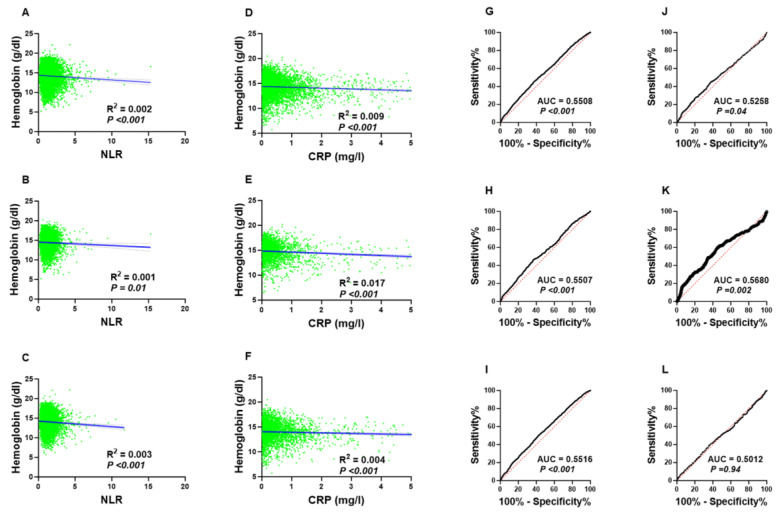
Association and diagnostic performance of NLR and CRP for anemia: Simple linear regression of the association of NLR and Hb (**A**) in both genders, (**B**) in males, and (**C**) in females. Simple linear regression of the association between CRP and Hb (**D**) in both genders, (**E**) in males, and (**F**) in females. ROC curves of NLR to discriminate anemics (**G**) in both genders, (**H**) in males, and (**I**) in females. ROC curves of CRP to discriminate anemics in (**J**) both genders, (**K**) males, and (**L**) females.

As shown in Figure 4, linear regression analysis in all subjects or within sex group found no correlation between NLR and Hb concentrations; yet, the association was statistically significant (Figure 4A–C). Likewise, the correlation between CRP and Hb followed a similar pattern. These findings suggest that while inflammatory markers do not directly correlate with Hb concentration, they might have an impact on Hb level indirectly with other variables directly involved in this process.

To further assess the clinical utility of NLR in differentiating anemic from non-anemic groups, we performed a receiver operating characteristic (ROC) curve analysis in both genders and in males and females. As shown in Figure 4G–I, the area under the curve (AUC) was almost the same (0.55), which is better than CRP (Figure 4J–L), specifically in the female group (AUC = 0.55 vs. AUC = 0.50).

## 4. Discussion

The present study was undertaken to demonstrate and establish the link between anemia and inflammation by examining the NLR in anemic and non-anemic individuals. The main finding in our study is that we showed for the first time that Saudi subjects with anemia exhibited a significant increase in NLR compared to the non-anemic group. Since it is well accepted that the inflammatory state is augmented in anemia, it is not surprising that NLR is elevated in anemic subjects regardless of age or gender. Notably, Singh et al. has demonstrated that in a small group of 20 patients with nutritional anemia, total leukocyte count was found to be higher in anemic patients compared to that of controls; yet, statistical significance was not achieved [20]. More importantly, NLR was significantly higher in anemics [20], which is in agreement with our current findings. Stratified by NLR, our study showed that subjects with increased NLR had a significantly lower Hb compared to those with normal NLR.

A number of studies have reported conflicting data regarding the relationship of inflammatory markers with anemic parameters. In patients on dialysis, high levels of CRP were associated with lower Hb [21]. Similarly, CRP concentration was significantly elevated in anemic neonates with acute kidney injury [22]. However, a marginal association between CRP and Hb in 16095 healthy individuals undergoing a regular medical examination was noted [23]. Likewise, a moderate linear association between Hb and CRP was reported in hemodialysis patients [24]. Additionally, it was reported that the differences in median levels of IL-6 and CRP between anemic and non-anemic elderly patients were relatively small [25]. In our study, we present a comparative analysis of NLR and CRP in light of anemia, which revealed that despite the fact that CRP is significantly elevated in anemics, it was a less sensitive marker of Hb status compared to NLR, suggesting that the overall performance of the latter is superior to CRP in discriminating anemics from non-anemics. Given that CRP is an acute-phase protein and a pattern-recognition marker of systemic inflammation, it seems plausible to assume that CRP is less susceptible to chronic, low-grade inflammation, which may explain its poor sensitivity to discriminate anemics in our study population.

In this report, sex-specific comparisons revealed that NLR failed to distinguish between anemic and non-anemic in young and elderly males but not females. This might be attributed to the ability of inflammatory cells to mount an immune response against inflammation-associated anemia. It is well known that the magnitude as well as the speed of immune responses elicited against pathogens can dramatically differ between the sexes [26]. In general, females mount stronger and faster innate and adaptive immune responses than males [27]. Moreover, women of reproductive age are more vulnerable to anemia due to blood loss associated with menstruation, childbirth, as well as hemodilution during pregnancy [28]. These factors might partially account for the gender disparity associated with changes in NLR in relation to anemia.

Although the diagnostic value of NLR in anemia was poor in this study, others have nonetheless shown the usefulness of NLR in predicting the development and progression of anemia associated with chronic diseases. NLR was examined in sickle cell anemia and was found to be high with proteinuria and positively correlated with fibrinogen levels [29]. Furthermore, NLR and anemia were independent risk factors for mortality in breast cancer patients [30]. Elevated NLR combined with decreased Hb predicted the onset of major adverse cardiovascular events in patients with ST-elevation myocardial infarction [14]. Taken together, it appears that NLR is most appropriate for following up and managing anemia associated with chronic disease rather than screening or diagnosing anemia at the population level; yet, further longitudinal studies are certainly warranted.

There are limited studies addressing the usefulness of other inflammatory ratios including monocyte–lymphocyte ratio (MLR), platelet–lymphocyte ratio (PLR), and systemic immune-inflammation index (SII) in light of anemia. The fact that inflammatory ratios can be easily determined from routine blood work makes it a simple and inexpensive addition to the patient work-up. These inflammatory ratios have been examined in various pathological conditions such as chronic kidney disease, metabolic syndrome, cancer, and COVID19 infection [31,32,33,34,35]. It has recently been shown that when compared to other inflammatory ratios, SII was a more reliable predictor of surgical trauma in elderly patients with hip fractures [36]. More studies are needed to characterize patterns of CBC-derived inflammatory ratios in relation to chronic diseases such as anemia.

The association of hypoxia and ischemia with anemia might trigger inflammatory cascades in leukocytes by increasing vascular reactivity to catecholamines [37]. Furthermore, iron has been shown in many studies to be crucial for the proliferation and maturation of immune cells, particularly lymphocytes, which is associated with the specific response to infection [38]. Although the exact mechanism is not well defined, hepcidin has been proposed as a link between iron availability and immune response [39]. Hepcidin expression is upregulated by inflammation and it has also been shown that interleukin-6 enhances the hepatic synthesis of hepcidin, which regulates iron recycling, leading to the development of anemia due to hypoferremia [40].

Our study is not without limitations. First, the causality between NLR and anemia could not be established. Second, data on potential confounding variables, such as body mass index, physical activity, and smoking, are lacking. Nevertheless, our study has numerous advantages, most notably the very large sample size and efficient data collection. Further prospective studies are needed in order to examine the association and causality between inflammation and anemia.

## 5. Conclusions

In conclusion, our study demonstrated that elevated NLR is consistently observed in anemic subjects and carries a greater risk for anemia. These findings reflect the roles of neutrophils and lymphocytes in the constitutive inflammatory process. Due to its simplicity, NLR should be used more frequently in the routine clinical assessment of anemia. However, since NLR lacks sensitivity, further research is certainly required to assess its effectiveness in predicting the risk of anemia.

## Figures and Tables

**Table 1:** Age distribution of study subjects.

	Number of Subjects (%)	Mean WBC Count (×10^6^/mL) (95% CI)	Mean NLR (95%CI)
***Male***			
Young	3.05	6.17 (5.99–6.35)	1.25 (1.19–1.31)
Young Adult	16.65	6.06 (5.99–6.14)	1.26 (1.23–1.29)
Adult	18.21	6.01 (5.94–6.08)	1.23 (1.21–1.26)
Elderlies	3.93	6.17 (6.01–6.32)	1.31 (1.12–1.37)
***Female***			
Young	3.02	5.93 (5.74–6.12)	1.15 (1.08–1.22)
Young Adult	30.36	5.98 (5.93–6.04)	1.24 (1.22–1.26)
Adult	20.12	6.04 (5.97–6.11)	1.26 (1.24–1.29)
Elderlies	4.39	6.02 (5.87–6.18)	1.24 (1.18–1.29)
***Unknown gender***	0.27	6.11 (5.46–6.76)	1.26 (1.10–1.41)

**Table 2:** Prevalence of non-anemic and anemic relative to either NLR or CRP.

Parameter	Non-Anemic	Anemic
***Both genders***		
N-NLR	97.33	95.02
H-NLR	2.67	4.98
N-CRP	85.05	79.08
H-CRP	14.95	20.92
***Male***		
N-NLR	97.63	95.54
H-NLR	2.37	4.46
N-CRP	86.73	75.38
H-CRP	13.27	24.62
***Female***		
N-NLR	97.09	94.72
H-NLR	2.91	5.28
N-CRP	83.24	80.93
H-CRP	16.76	19.07

**Table 3:** Risk assessment of elevated NLR and anemia.

	Score	95% CI	z Statistic	Significance Level
**PR**				
Both genders	1.87	1.46–2.40	4.92	*p* < 0.0001
Males	1.88	1.23–2.89	2.89	*p* = 0.004
Females	1.81	1.34–2.46	3.83	*p* = 0.0001
**OR**				
Both genders	1.91	1.47–2.48	4.88	*p* < 0.0001
Males	1.92	1.23–3.01	2.87	*p* = 0.004
Females	2.22	1.64–3.00	5.18	*p* < 0.0001

**Table 4:** Risk assessment of elevated CRP and anemia.

	Score	95% CI	z Statistic	Significance Level
**PR**				
Both genders	1.40	1.18–1.65	3.95	*p* = 0.0001
Males	1.86	1.42–2.42	4.53	*p* < 0.0001
Females	1.14	0.92–1.42	1.19	*p* = 0.24
**OR**				
Both genders	1.51	1.22–1.85	3.85	*p* = 0.0001
Males	2.13	1.51–3.02	4.28	*p* < 0.0001
Females	1.17	0.90–1.52	1.18	*p* = 0.24

## Data Availability

Data are available from the corresponding author upon reasonable request, and with permission of Al Borg Diagnostics.
